# Cystic transformation of focal lesions after therapy is associated with remission but adverse outcome in myeloma

**DOI:** 10.1038/s41408-019-0235-3

**Published:** 2019-08-27

**Authors:** Maximilian Merz, Thomas Hielscher, Elias Karl Mai, Anja Seckinger, Dirk Hose, Anna Jauch, Sandra Sauer, Steffen Luntz, Uta Bertsch, Marc S. Raab, Kai Neben, Hans Salwender, Igor W. Blau, Hans-Walter Lindemann, Jan Dürig, Christof Scheid, Mathias Haenel, Katja Weisel, Tim Weber, Stefan Delorme, Hartmut Goldschmidt, Jens Hillengass

**Affiliations:** 1 Department of Internal Medicine V, University Hospital Heidelberg, Heidelberg, Germany; 2 Roswell Park Comprehensive Cancer Center, Buffalo, NY USA; 3 Division of Biostatistics, German Cancer Research Center (DKFZ), Heidelberg, Germany; 4 Institute of Human Genetics, University Heidelberg, Heidelberg, Germany; 5 Coordination Center for Clinical Trials, University Hospital Heidelberg, Heidelberg, Germany; 6 National Center for Tumor Diseases (NCT), Heidelberg, Germany; 7 Max-Eder Research Group Experimental therapies for hematologic malignancies, DKFZ, Heidelberg, Germany; 8 Asklepios Klinik und St. Georg, Altona, Hamburg, Germany; 9 Department of Internal Medicine III, Charité Campus Benjamin Franklin, Berlin, Germany; 10 Department of Hematology and Oncology, Kath. Krankenhaus Hagen gem. GmbH - St.-Marien-Hospital, Hagen, Germany; 11 Department of Hematology and Oncology, University Hospital of Essen, Essen, Germany; 12 Department of Internal Medicine I, University of Cologne, Cologne, Germany; 13 Department of Internal Medicine III, Klinikum Chemnitz gGmbH, Chemnitz, Germany; 14 II. Medizinische Klinik und Poliklinik, Universitätsklinikum Hamburg-Eppendorf (UKE), Hamburg, Germany; 15 Department of Radiology, University Hospital Heidelberg, Heidelberg, Germany; 16 Department of Radiology, German Cancer Research Center DKFZ, Heidelberg, Germany

**Keywords:** Translational research, Myeloma, Cancer imaging

Dear Editor,

Response assessment in multiple myeloma (MM) is based on measurements of monoclonal proteins and minimal residual disease (MRD) in bone marrow samples from the iliac crest^1^. Since discrete areas of plasma cell accumulation can be visualized as focal lesions (FL) by magnetic resonance imaging (MRI) as well as positron-emission computed tomography (PET/CT), the International Myeloma Working Group (IMWG) has included MRI and PET/CT in their updated guidelines for primary diagnosis and follow-up^1,2^. We analyzed conventional MRI at primary diagnosis and after ASCT in newly diagnosed patients enrolled in the prospective MM5 trial (EudraCT No. 2010-019173-16) of the German-Speaking Myeloma Multicenter Group (GMMG). Treatment of newly diagnosed, symptomatic MM patients within the GMMG MM5 trial consisted of three cycles PAd (bortezomib, doxorubicin, dexamethasone) or VCD (bortezomib, cyclophosphamide, dexamethasone) induction therapy; high dose melphalan followed by ASCT as well as consolidation and maintenance therapies with lenalidomide for 2 years or until complete response (CR) (Supplemental Fig. 1). For patients not achieving a near complete response (nCR) or CR after the first ASCT a second ASCT was recommended. The study was performed in accordance with the Declaration of Helsinki, the European Clinical Trial Directive (2005) and was approved by the local ethics committees. Inclusion and exclusion criteria as well as primary end points of the study have been reported^3–5^. MRI was performed at primary diagnosis and inclusion into the GMMG MM5 trial and repeated after the last ASCT before the start of consolidation therapy (Supplemental Fig. 1). Whole-body imaging was performed using unenhanced T1-weighted turbo-spin echo sequences as well as T2-weighted short-tau inversion recovery (STIR) sequences and analyzed as described previously^6^. All images were assessed by two experienced investigators blinded to outcome. In accordance with the IMAJEM study of the IFM and previous analyses from our group, response to treatment was defined as signal decrease in T2-weighted images as well as signal recovery in T1-weighted images^7,8^. At inclusion into the trial, interphase fluorescence in situ hybridization (iFISH) and gene expression profiling (GEP) were performed on CD138-purified plasma cells to identify cytogenetic abnormalities as well as proliferation activity, as described previously^9,10^. Description of statistical analyses can be found in the supplemental material. A total of 167 patients were enrolled in the GMMG MM5 trial at the University Hospital Heidelberg between July 2010 and October 2012. Eighty-three of the 167 patients received a MRI before starting the therapy, and 77 patients after the last ASCT before consolidation treatment (median 98 days after last ASCT, interquartile range: 20 days). Thirty-four patients (44.2%) were treated with tandem ASCT and at the time of the second MRI, 41 patients (53.2%) had achieved a near CR/CR. Patient characteristics are summarized in supplemental table 1. FL were found in 76 patients (91.6%) at primary diagnosis, diffuse marrow infiltration in 81 patients (97.6%). Disease growing beyond cortical bone was detected in 21 patients (25.3%). After ASCT, residual FLs were found in 62 patients (80.5%), and residual diffuse infiltration in 47 patients (61.0%). Response to treatment of FL is usually characterized by signal normalization in T1- as well as T2-weighted images. However, in 28 patients (45.2% of patients with residual FLs) we observed in a subset of FL a cystic transformation after ASCT. Cystic transformation of FL was characterized by signal intensity similar to cerebrospinal fluid on T2- and T1-weighted images (Fig. 1). When analyzing cystic FL after therapy, no distinct anatomical location, no morphologic feature or size at baseline could be identified that would have predicted cystic transformation. In 4 of the 28 patients with cystic FL a PET/CT had been performed at the same time point. None of the respective lesions showed increased FDG uptake (Fig. 1). No significant associations between the presence of residual FLs and diffuse infiltration with rates of nCR/CR after ASCT were observed. Patients with residual FLs and a cystic transformation showed significantly higher rates of nCR/CR (75%) compared to patients without the respective changes after therapy (41%, *p* = 0.005). Analyses of baseline characteristics revealed that patients with cystic lesions after therapy harbored more often a deletion of chromosome 13q14 (61.5 vs. 33.3%, *p* = 0.03), disease exceeding bone (48.0 vs. 7.1%, *p* < 0.001) as well as a medium/high proliferation index as assessed by GEP at baseline (92 vs. 67%, *p* = 0.03). No associations between MRI findings and the other tested cytogenetic abnormalities were found, especially not for high-risk abnormalities (del17p, *t*(4; 14), gain1q21) or a hyperdiploid karyotype. Presence of bone-exceeding disease at baseline was associated with shorter OS (5 year OS 59%, 95% confidence interval (CI) [40%; 87%] vs 83% [73%; 93%], *p* log-rank: 0.03, Fig. 2b) while no significant differences for PFS were found (Fig. 2a). When analyzing the entire cohort, the disappearance or persistence of FLs or diffuse infiltration was not associated with significant survival differences. However, patients not achieving a CR after ASCT had a shorter PFS (Fig. 2c) if diffuse marrow infiltration was present at the second MRI (Median PFS: 26 months 95%CI [24; 34] vs. 54 months [32, not reached], *p* log-rank: 0.03). Also, patients with cystic transformation of FLs after therapy had a shorter PFS than patients without such signal alterations (Median PFS: 17 months 95%CI [14; 34] vs. 45 months [29, not reached], *p* log-rank: 0.014, Fig. 2e), but this did not apply for OS which was identical for both groups (Fig. 2f). The presence of cystic lesions retained their negative prognostic impact after adjustment for ISS, treatment arm, performance of single or tandem ASCT as well as response after ASCT (Hazard ratio, 95% CI: 2.47 [1.25; 4.91], *p* = 0.0097). Changes of MRI before and after ASCT have been associated with outcome in retrospective analyses of newly diagnosed MM^8^. However, the recent prospective IMAJEM study by the Intergroupe Francophone du Myélome showed for the first time that return of MRI to normal after ASCT does not allow predicting outcome^7^. Normalization of MRI is rather infrequent after therapy. It occurred in 11%, 13%, and 20% in the IMAJEM trial, a previous analysis of our group and the current study, respectively^7,8^. In the current analysis, residual FL or diffuse infiltration were not associated with adverse outcome when analyzing the entire cohort, which is also in line with the IMAJEM trial^7^. Additionally, we demonstrated that the presence of disease exceeding cortical bone is a negative prognostic factor, even in the era of proteasome inhibitor-based induction therapy and lenalidomide maintenance^7,11^. Furthermore, we observed that almost half of our patients with residual FL showed a mixed pattern of signal alterations. In general, signal intensity of responding FL or diffuse infiltration would be expected to level up with the surrounding unaffected bone marrow. However, T2 hyperintense transformation upon successful therapy has been described especially for bone metastases from solid tumors^12^. Liquefaction of necrotic tumor tissue is thought to be the reason for this effect that might mimic active disease also in diffusion-weighted images (DWI). In order to confirm the hypothesis that the observed effects were caused by rapid tumor cell decay in proliferative disease, we analyzed baseline characteristics of patients who after therapy would have cystic FL. We found that patients with such changes were less likely to harbor slowly proliferative disease as assessed by GEP from random bone marrow aspirates. Our assumption was furthermore supported by the fact that patients with cystic FL achieved higher rates of nCR/CR after ASCT and none of the lesions showed FDG uptake in the small group of patients with available PET/CT at the same time point. Regardless of deep remissions after ASCT, patients with cystic lesions showed shorter PFS, even after adjustment for treatment and response. We hypothesize that these findings also in part explain the adverse prognosis of patients who lose an initially deep response shortly after high dose chemotherapy^13^. However, no significant effect on OS was observed, which might be a result of the limited number of patients or limited follow-up. Additionally, patients without CR after ASCT and residual diffuse MRI signal alterations had an adverse outcome. Diffuse bone marrow infiltration as detected by MRI at baseline has been associated with high-risk disease and adverse outcome after ASCT^14,15^. Importantly, patients not achieving a CR upon treatment within the GMMG MM5 trial were treated with lenalidomide maintenance for 2 years. This explains the separation of PFS curves at approximately 24 months and might support longer or even continuous treatment in patients with suboptimal response and residual diffuse infiltration in MRI. To address limitations of the current study in future trials, findings from MRI need to be correlated with PET/CT in a larger number of patients to confirm that cystic FL are uniformly PET negative. Furthermore, imaging findings should be correlated to MRD assessment to clarify the discrepancies between PET/CT and MRD negativity by flowcytometry found in the IMAJEM trial by the IFM^7^.

**Fig. 1:**
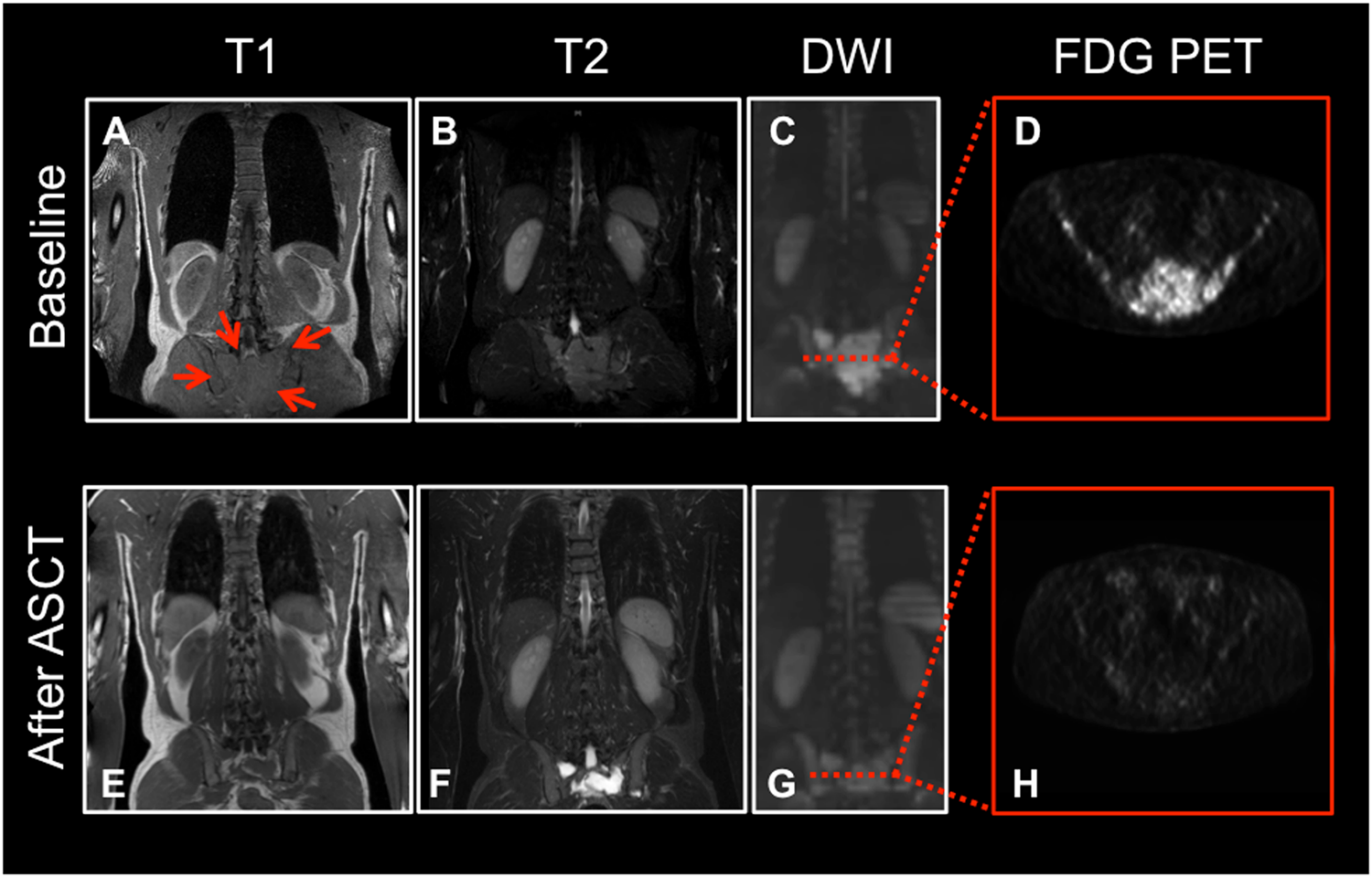
Cystic transformation of FL. Representative images from a patient with a large focal lesion in the sacrum with extramedullary growth. Images at baseline in the top row with common features of a focal myeloma lesion (red arrows): Decreased signal intensity on T1-weighted images (**a**) and increased intensity on T2-weighted images (**b**) as well as DWI (**c**). PET/CT proved increased FDG uptake at primary diagnosis. Upon achieving remission after ASCT (bottom row), the lesion decreased significantly in size as depicted on T1- (**e**) and T2-weighted (**f**) images. The lesion showed a hyperintense signal on T2-weighted images but no increased FDG uptake compared to the surrounding tissue. MRI images in coronar orientation, PET/CT in transversal orientation

**Fig. 2:**
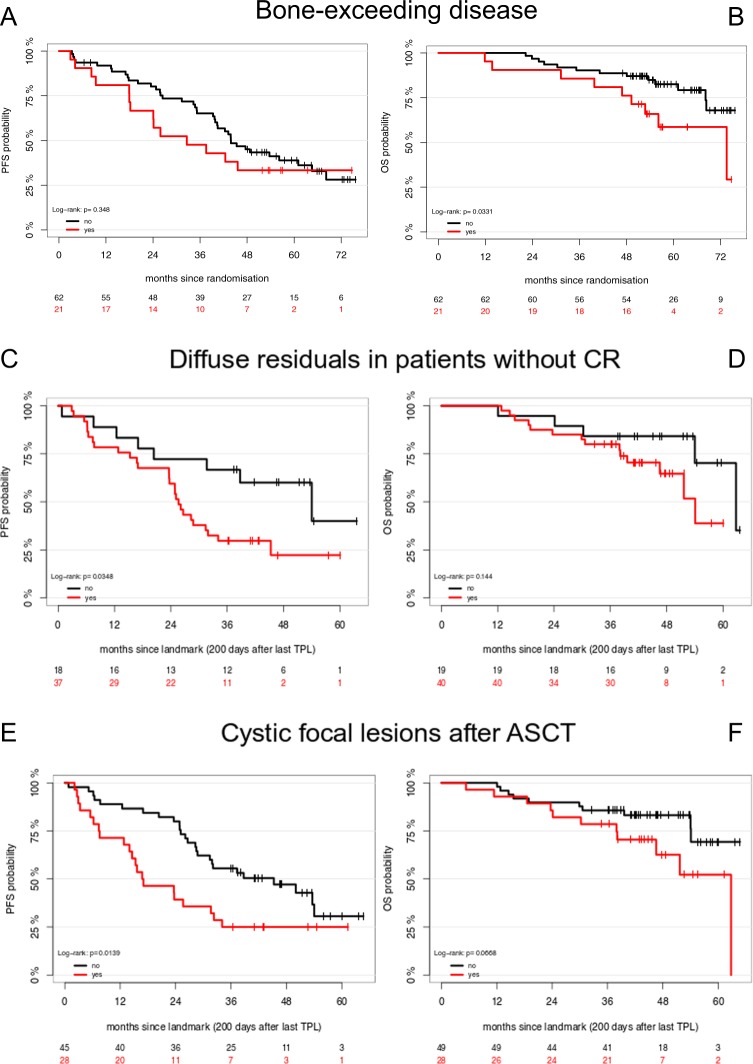
Survival according to MRI findings before and after ASCT. Progression-free (PFS) and overall survival (OS) analysis in patients with bone-exceeding disease (**a** and **b**) as well as patients not achieving CR and with residual diffuse infiltration after ASCT (**a** and **b**). PFS (**c**) and OS (**d**) for patients with cystic lesion after ASCT

In summary, return of MRI findings to normal was of prognostic significance in patients without CR after ASCT. MRI identifies a subgroup of patients with high-quality responses but adverse outcome after therapy.

## Supplementary information

Supplemental material
