# Supplementary material for: Cystic transformation of focal lesions after therapy is associated with remission but adverse outcome in myeloma
Source: Blood Cancer J. 2019 Aug 27;9(9):71. doi: 10.1038/s41408-019-0235-3 (PMC6712022; doi:10.1038/s41408-019-0235-3)

**Merz et al.**

***Cystic transformation of focal lesions after therapy is associated with remission but adverse outcome in myeloma***

**Supplemental material**

**Statistical analysis**

Patients were excluded if the first MRI had been performed more than 28 days after treatment start, and so were patients with a second MRI more than 200 days after last ASCT or a second MRI after progression. Response assessment closest to the second MRI but within a 60 days time window was used for correlation between remission status and MRI findings. Progression-free survival (PFS) was defined as time from randomization to progression or death, whichever occurred first. Overall survival (OS) was defined as time from randomization to death from any cause. For prognostic impact of MRI parameters at 2nd MRI, PFS and OS were calculated from landmark time 200 days after last ASCT. Fisher’s exact test was used to compare categorical parameters. Kaplan-Meier method and log-rank test were used to analyze distribution of survival times. A multivariable Cox regression model was used to account for clinical parameters. . All p-values were two-sided. P-values <0.05 were considered significant. Analyses were performed using R software (version 3.2.).

**Supplemental** **Table 1. Patient characteristics**

|  | **level** | **n** | **%** |
| --- | --- | --- | --- |
| **ISS** | I | 41 | 49.4 |
|  | II | 24 | 28.9 |
|  | III | 18 | 21.7 |
| **Induction** | PAd | 41 | 49.4 |
|  | VCD | 42 | 50.6 |
| **Lenalidomide maintenance** | for 2 years | 46 | 55.4 |
|  | until CR | 37 | 44.6 |
| **Remission** | CR/nCR | 41 | 53.3 |
|  | VGPR / PR / MR | 35 | 45.5 |
| **MRI before ASCT** | | | |
| **Focal lesions** | Yes | 76 | 91.6 |
|  | No | 7 | 8.4 |
| **Diffuse** | Yes | 81 | 97.6 |
|  | No | 2 | 2.4 |
| **Bone exceeding** | Yes | 21 | 25.3 |
|  | No | 62 | 74.4 |
| **MRI after ASCT** | | | |
| **Focal lesions** | Yes | 62 | 80.5 |
|  | No | 15 | 19.5 |
| **Diffuse** | Yes | 47 | 61.0 |
|  | No | 30 | 39.0 |

ISS, International Staging System

PAd, bortezomib / doxorubicin / dexamethasone

VCD, bortezomib / cyclophosphamide / dexamethasone

CR, complete response

nCR, near complete response

VGPR, very good partial remission

PR, partial remission

MR, minimal response

MRI, magnetic resonance imaging

**Supplemental Figure 1. Flow chart of GMMG MM5 study**

Patients were randomized to 3 cycles PAd (bortezomib, doxorubicin, dexamethasone) or VCD (bortezomib, cyclophosphamide, dexamethasone) induction therapy and maintenance therapies with lenalidomide for 2 years or until complete response, resulting in 4 different treatment arms. MRI was performed at study inclusion as well as after ASCT before the start of consolidation therapy


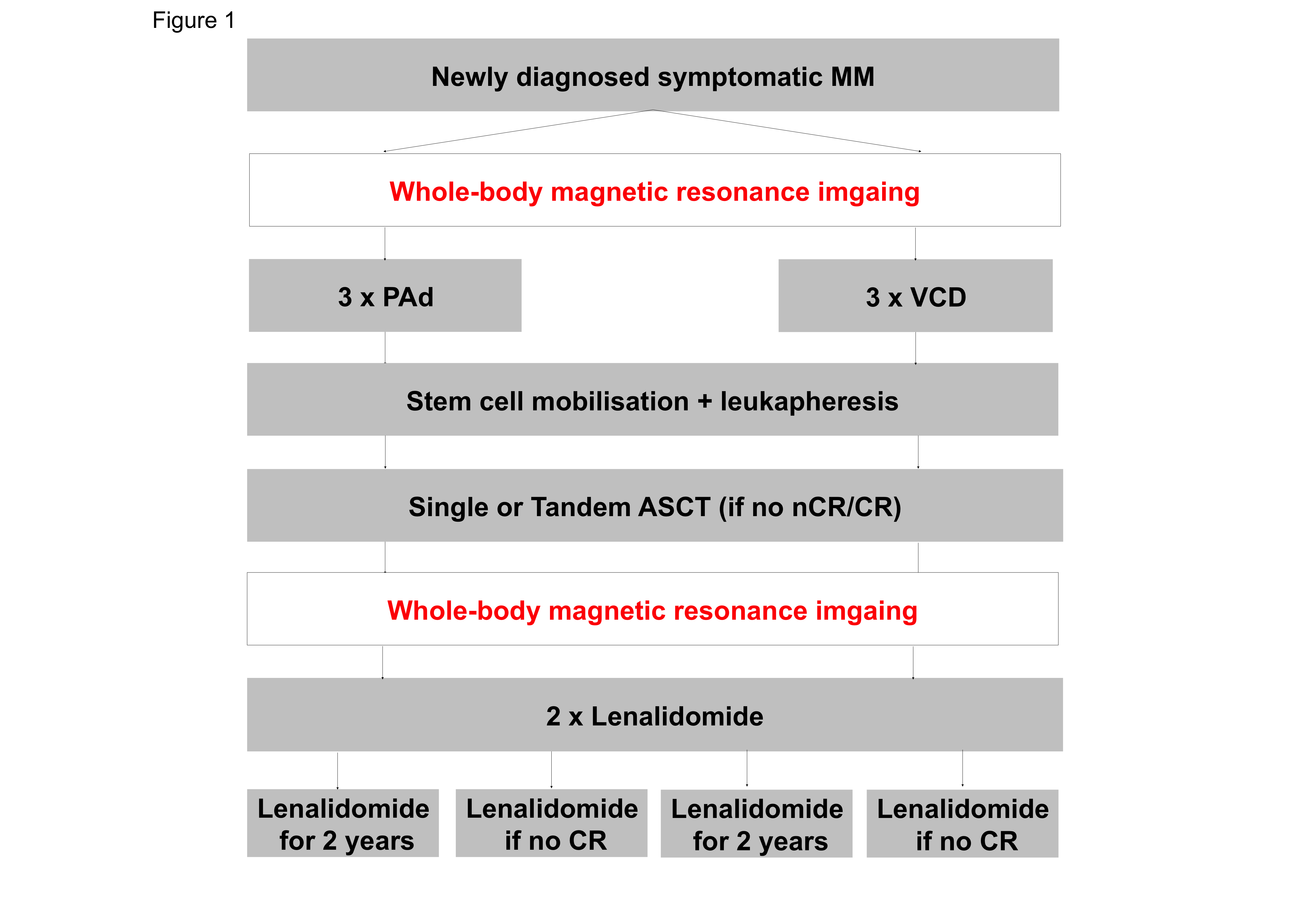

Supplement: Supplementary file 1 — Supplemental material [file 41408_2019_235_MOESM1_ESM.docx]
